# Supplementary figures and images for: Hyperbolic odorant mixtures as a basis for more efficient signaling between flowering plants and bees
Source: PLoS One. 2022 Jul 13;17(7):e0270358. doi: 10.1371/journal.pone.0270358 (PMC9278781; doi:10.1371/journal.pone.0270358)

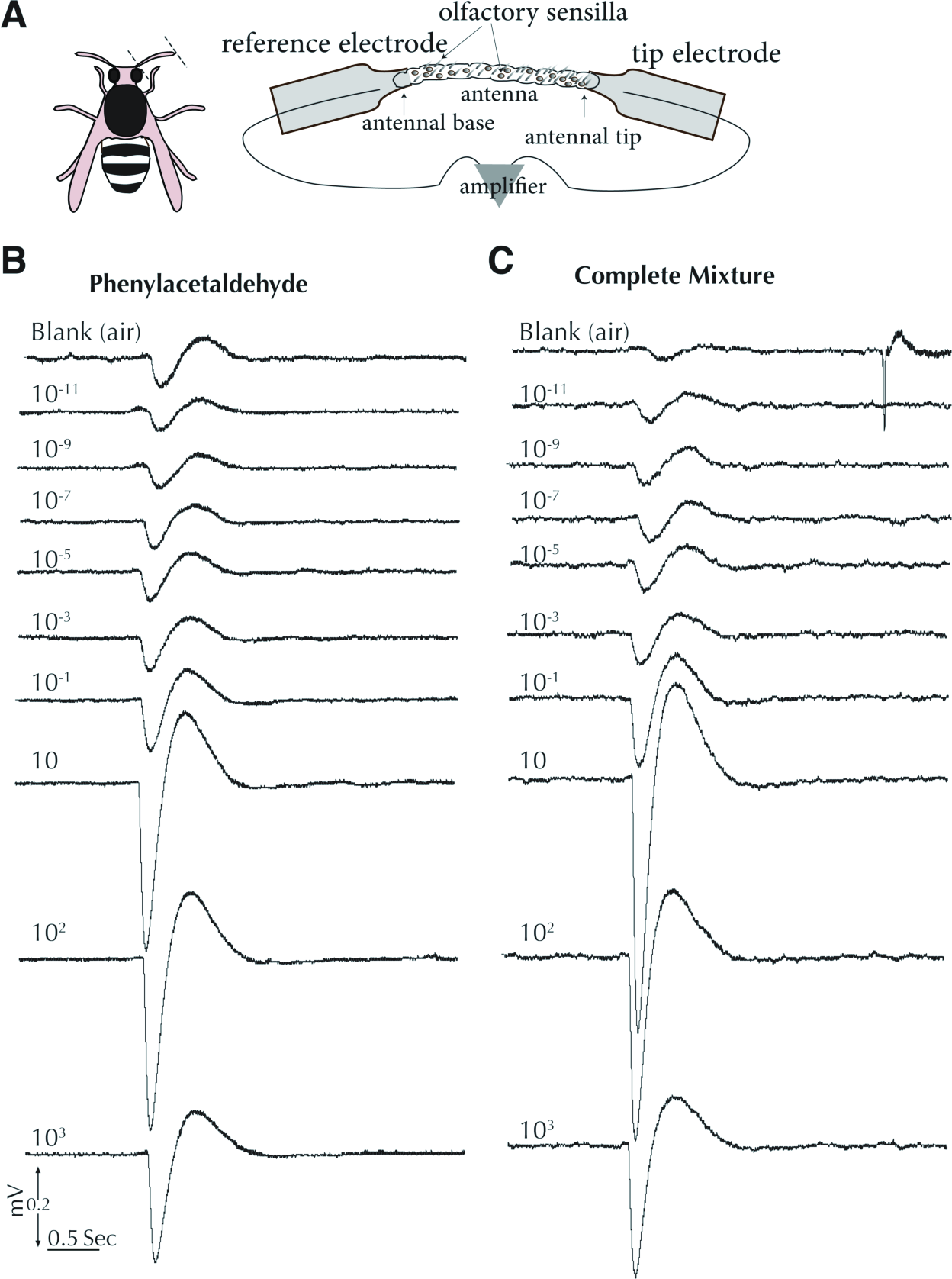

Supplement: S1 Fig — (A) a schematic drawing of an electroantennaography setup. The two ends of the antennal shaft, after being cut off from a bee’s head capsule, were inserted in the two capillary glasses (reference and tip electrodes) containing insect ringer’s solution. For more details about the procedure see Method Details. Sample electroantennogram traces when a honey bee was exposed to the increasing concentrations of (B) phenylacetaldehyde and (C) complete mixture. Note that the olfactory responses to both stimuli at 102 and 103 g/L are lower than those at 10 g/L. The responses are restored after enough inter-delivery time was given to the OSNs (see Fig 1). (TIF) [file pone.0270358.s001.tif]

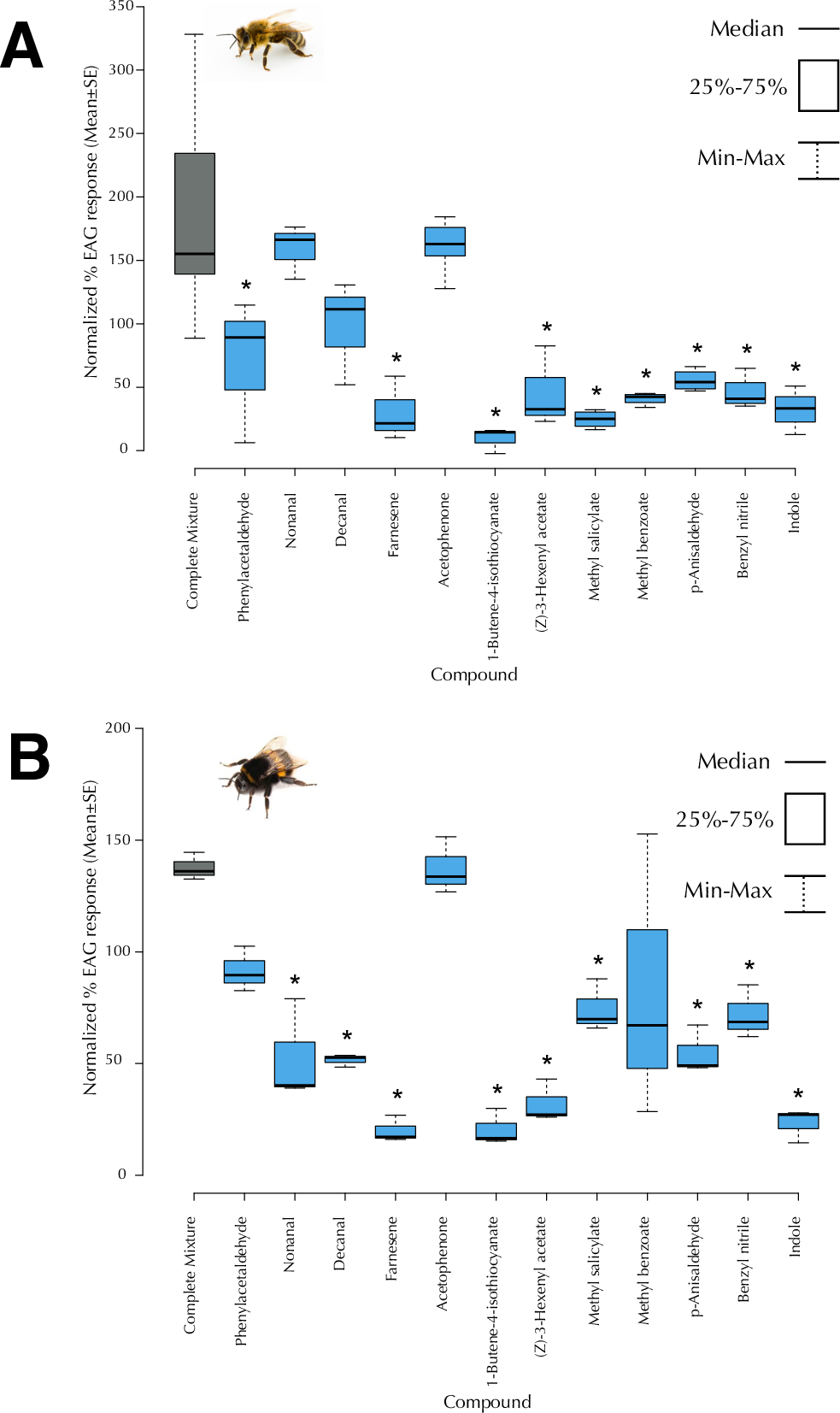

Supplement: S2 Fig — Electroantennographic responses of (A) honey bees (n = 3) and (B) bumble bees (n = 3) to the scent of Brassica rapa at 10 g/L. We first compared the intensity of the responses obtained when the compounds were presented individually at 10 g/L and found that the EAG values for the different odors in both species are significantly different (ANOVA, FApis = 9.86, FBombus = 11.16, df = 12, p<0.00001). We then compared the intensity of the responses obtained when the compounds were presented individually at 10 g/L with that in the complete mixture. The EAG values for the different odors in both species are significantly different from those for the mixture (ANOVA, df = 12, Tukey’s HSD, p<0.05). Nonetheless, in Apis mellifera the response intensity to nonanal, decanal, and acetophenone alone increased to that elicited by the mixture (Tukey’s HSD, p>0.1). In Bombus terristris, however, the response to phenylacetaldehyde, acetophenone, and methyl benzoate was similar to the mixture (Tukey’s HSD, p>0.1). (TIF) [file pone.0270358.s002.tif]

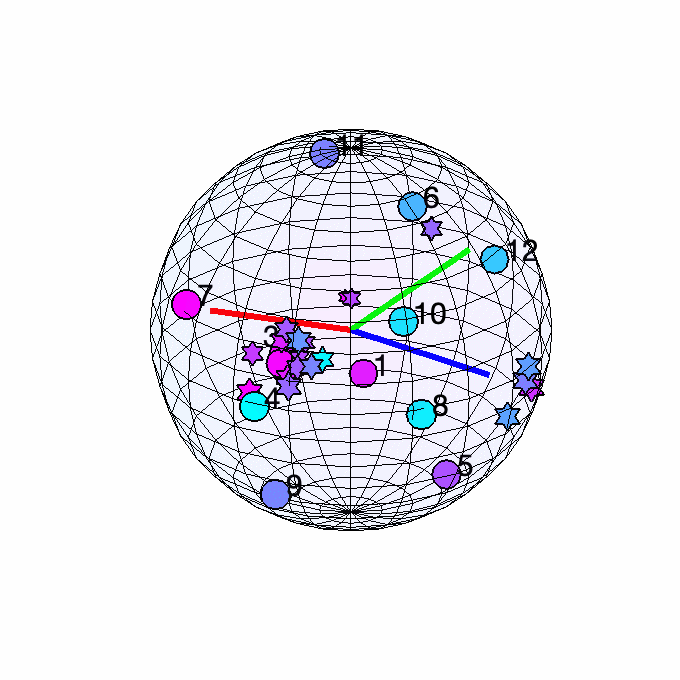

Supplement: S3 Fig — (GIF) [file pone.0270358.s003.gif]
